# Three-Dimensionally Printed K-Band Radar Stealth Lightweight Material with Lotus Leaf Structure

**DOI:** 10.3390/polym16182677

**Published:** 2024-09-23

**Authors:** Chuangji Liu, Yingjie Xu, Beiqing Huang, Wan Zhang, Yuxin Wang

**Affiliations:** College of Printing and Packaging Engineering, Beijing Institute of Graphic Communication, No. 1 (Band-2) Xinghua Street, Beijing 102600, China; liuchuangji1106@163.com (C.L.); huangpeiqing@bigc.edu.cn (B.H.); zhangwan@bigc.edu.cn (W.Z.); c2206366425@163.com (Y.W.)

**Keywords:** K-band radar stealth, UV-cured polymer composites, 3D printing technology, biomimetic lotus leaf antireflection structure, self-cleaning surface, lightweight material

## Abstract

K-band radar waves have high penetration and low attenuation coefficients. However, the wavelength of this radar wave is relatively short; thus, designing and preparing both broadband and wide-angle radar wave absorbers in this band presents considerable challenges. In this study, a resin-based K-band radar wave absorber with a biomimetic lotus leaf structure was designed and formed by UV curing. Here, microscale lotus leaf papillae and antireflection structures were prepared using a DLP 3D printer, and the contact angle between the material and water droplets was increased from 56° to 130°. In addition, the influence of the geometric parameters of the lotus leaf antireflection structure on the electromagnetic absorption performance and mechanical strength was investigated. After simulation optimization, the maximum electromagnetic loss of the lotus leaf structure 3D-printed sample was −32.3 dB, and the electromagnetic loss was below −10 dB in the 20.8–26.5 GHz frequency range. When the radar incidence angle was 60°, the maximum electromagnetic loss was still less than −10 dB. The designed lotus leaf structure has a higher mechanical energy absorption per unit volume (337.22 KJ/m^3^) and per unit mass (0.55 KJ/Kg) than commonly used honeycomb lightweight structures during the elastic deformation stage, and we expect that the designed structure can be used as an effective lightweight material for K-band radar stealth.

## 1. Introduction

Radar stealth technology is widely used in the military field; however, with ongoing development of radar detection technology, the survival and penetration ability of weapons and equipment in modern warfare are facing severe challenges. The key to enhancing the attack and defense capabilities of weapon systems is to reduce radar wave reflections effectively [1,2]. The development trend in radar wave absorbers is focused on strong electromagnetic absorption ability, light materials, thin structures, and good environmental adaptability [3,4]. K-band radar systems use K-band electromagnetic waves for detection and measurement. The frequency range of K-band electromagnetic waves is 18–27 GHz, with high penetration and low attenuation coefficients. These waves can pass through weather interference, e.g., clouds, rain, and snow, and K-band radar is suitable for long-distance detection and measurement purposes [5,6,7,8].

The stealth of K-band radar is realized by reducing the radar cross section of weapon equipment [9,10,11]. Here, there are two main approaches. One approach uses coating materials that can absorb radar waves, and the other approach involves changing the structure and shape of the radar-absorbing material to construct an antireflection structure. Typically, such structures are undulating, and when radar waves pass through their surfaces, a series of refraction and internal reflection processes occur at the interface, which increases the path length of the incident radar waves and reduces the reflectivity of the material’s surface [12,13]. The high electromagnetic loss of radar wave-absorbing materials under oblique incidence is also being studied. Radar waves are generally obliquely incident; thus, radar wave absorbers must possess good absorption characteristics for electromagnetic waves that are incident at a wide angle. For electromagnetic waves with different incident directions, radar wave absorbers must exhibit isotropy, which requires the size of the material’s basic unit structure to be subwavelength [14,15,16]. For K-band radar absorbers, the size of their basic unit structure is a few millimeters to a few centimeters, which poses a considerable challenge in the material manufacturing process.

3D printing technology, also referred to as additive manufacturing, has received increasing research attention for the preparation of structural radar wave absorbers [17,18,19,20,21,22,23,24,25]. For example, Schmitz et al. prepared honeycomb structures based on 3D-printed polylactic acid, which has strong absorption intensity and wide absorption frequency band for radar waves in the X-band and Ku band [26], and Li et al. prepared a novel honeycomb-hollow pyramid sandwich structure using a 3D printer, which exhibits good absorption performance for electromagnetic waves with an incident angle of 45° [27]. In addition, Chen et al. 3D-printed a pyramid absorber that can absorb electromagnetic waves effectively with polarization angles less than 50°–60° [28]. Phan et al. prepared an absorber comprising a periodically truncated cone water-filled container using a 3D printer, and this absorber has a wide incidence angle and polarization insensitivity at high temperatures [29]. Duan et al. prepared a complex gradient composite using 3D printing technology that exhibits good absorption for radar waves with incident angles less than 55° [30], and Warski et al. 3D-printed composites containing high-entropy ferrites with unique magnetodielectric properties, and these materials exhibit excellent electromagnetic absorption performance [31].

**Figure 1:**
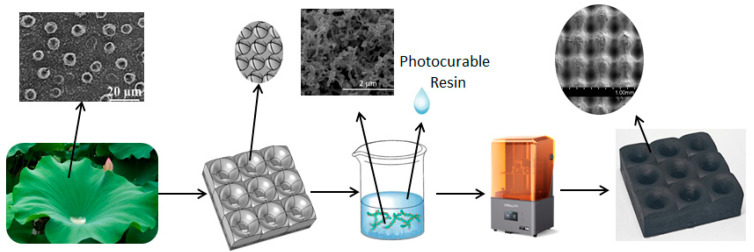
Schematic diagram illustrating the preparation of the biomimetic lotus leaf structure radar wave absorber.

Nature-inspired designs for biomimetic functional materials have resulted in the development of various biomimetic materials with special functions. For example, the shape of lotus leaves is a unique antireflection structure that can promote the absorption of radar waves effectively, and the micro/nanostructure on the surface of the lotus leaf endows it with a self-cleaning ability [32,33,34]. Design the biomimetic lotus leaf structure as a structural unit for radar wave absorption, enabling it to have both a wide incidence angle and self-cleaning ability. However, to prepare this structural unit, a 3D printer with micrometer-level printing accuracy is required [35]. Digital light processing (DLP) 3D printers are one of the highest precision 3D printers. The operating principle of these 3D printers is to irradiate an ultraviolet (UV) light source onto a photosensitive resin to trigger local polymerization and curing reactions, thereby forming a single-layer printing shape. Then, the movement of the printing platform is controlled to realize 3D printing [36,37]. The printing screen resolution of the DLP 3D printer used in this study is 13,320 × 5120 pixels with a pixel size of 16.8 × 24.8 μm, which enables the preparation of microscale lotus leaf structures. The radar wave absorber with a biomimetic lotus leaf antireflection structure and self-cleaning surface was fabricated by printing UV-cured materials using a high-precision DLP 3D printer (Figure 1).

## 2. Materials and Methods

### 2.1. Materials Preparation

Multiwalled Carbon Nanotube/Polypyrrole Nanotube/Fe_3_O_4_ Composites (MWCNT/PPy/Fe_3_O_4_ is synthesized in the laboratory) [38]. 4-acryloyl morpholine (ACMO), aliphatic polyurethane acrylate (Shanghai Yinchang New Materials Co., Ltd., Shanghai, China). 2,4,6-trimethylbenzoyl diphenyl phosphine oxide (TPO) (Tianjin Jiuri New Materials Co., Ltd., Tianjin, China). Defoamer 920, dispersant 670 (Evonik Industries AG, Essen, Germany).

A total of 500 mg of acidified MWCNTs was subjected to ultrasonic agitation in 50 mL of ethanol for 20 min. Next, the mixture was transferred to a three-necked flask placed in an ice-water bath, and 1 mL of pyrrole monomer was added. After stirring mechanically for 20 min, a solution containing 16.2 g of FeCl_3_·6H_2_O dissolved in 50 mL of deionized water was added dropwise to the flask. The mixture was mechanically stirred for 12 h to obtain PPy-coated MWCNTs. The three-necked flask was placed in a 60 °C water bath, and NH_3_·H_2_O was gradually added until the pH was 8–10. The mixture was stirred at a constant temperature for 30 min, allowed to settle, and was then separated using a strong magnet. The sample was filtered, washed with deionized water and ethanol, and dried in a vacuum oven at 60 °C for 24 h to obtain the MWCNT/PPyNT/Fe_3_O_4_ composite samples. The theoretical weight ratios of Fe_3_O_4_, CNTs, and PPy are 65%, 12%, and 23%, respectively [38]. To prepare the 3D-printed photopolymerization resin, the ACMO and polyurethane acrylate were mixed and stirred at a mass ratio of 3:2. 0.5 wt% MWCNT/PPy/Fe_3_O_4_ absorbent, 1 wt% defoamer 920, and 1 wt% dispersant 670 were then added to the 3D printing photopolymerization resin. After mechanical stirring for 30 min, a high-speed grinder was used to grind the mixture for 90 min, and then 3 wt% photoinitiator TPO was added to the mixture. The 3D-printed microwave-absorbing material was obtained after stirring for 30 min.

**Figure 2:**
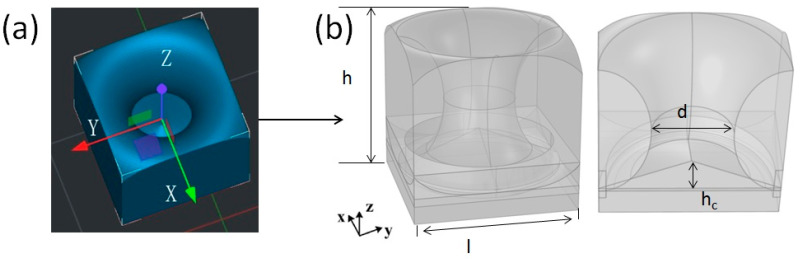
(**a**) Lotus leaf structure model and (**b**) design parameters.

In this study, a DLP UV-curable 3D printer (HALOT-MAGE S, Creality, Shenzhen, China) was used to fabricate the lotus leaf structure. Figure 2a shows a schematic diagram of the lotus leaf structure, which is a lightweight structure comprising lotus leaf-shaped hollow holes and a bottom cone. The geometric parameters of the lotus leaf structure are defined in Figure 2b. Here, l and h are the length and thickness of the lotus leaf structural units, respectively; d is the diameter at the narrowest point of the lotus leaf-shaped antireflection pore structure; and h_c_ is the height of the cone at the bottom of the antireflection structure.

To obtain the relative complex permittivity and complex permeability of the composite materials with the lotus leaf structure, samples with dimensions of 10.67 × 4.32 × 2.00 mm^3^ were prepared for K-band waveguide electromagnetic parameter testing. The data were imported into the CST Microwave Studio (2019) software to simulate and optimize the adsorption performance of the lotus leaf structures. Then, samples with dimensions of 180.00 mm × 180.00 mm × 10 mm were prepared using the DLP 3D printer based on the optimized geometric parameters for reflection loss testing. In addition, three units × two units of samples were prepared for compressive strength testing.

### 2.2. Characterization

The morphology and microstructure of the 3D printing materials were characterized using a field-emission scanning electron microscope (SEM, Hitachi, su8010, Chiyoda, Japan). The Raman spectra were measured on a Renishaw, UK, in via, and a vector network analyzer (VNA, N5234A PAN-L, Agilent, Santa Clara, CA, USA) equipped with waveguides (K-band) was employed to obtain the electromagnetic parameters of the printed samples. The predicted microwave absorption characteristics of the lotus leaf structure samples were calculated using Equations (1) and (2).

RL(dB) = 20log|(Z_in_ − 1)/(Z_in_ + 1)| (1)

Z_in_ = Z_0_(μ_γ_/ε_γ_)^1/2^tanh[j(2πfd/c)(μ_γ_ε_γ_)^1/2^] (2)

**Figure 3:**
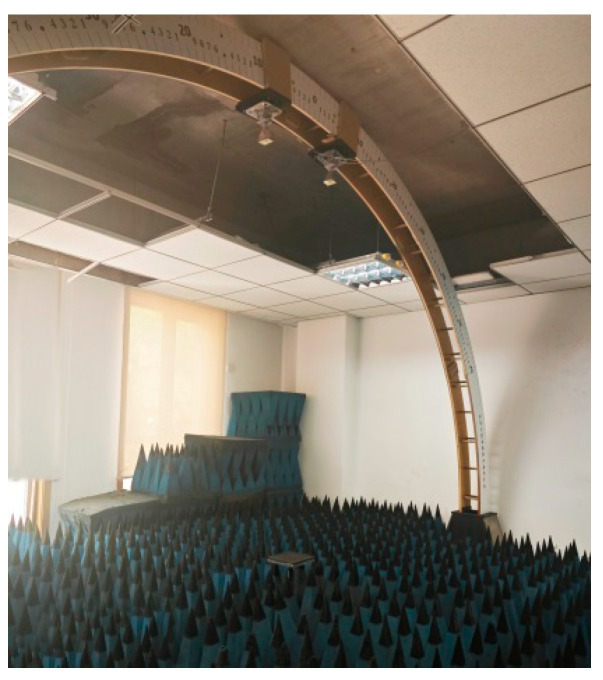
Arch-based reflection loss test system.

Here, Z_in_ and Z_0_ are the normalized input impedance of the absorbing material and the input impedance in free space, and μ_γ_ and ε_γ_ represent the relative magnetic permeability and dielectric constant of the material, respectively. In addition, d is the thickness of the absorbing layer, c is the speed of light, and f is the free space frequency of the electromagnetic waves [36]. The reflection loss of the lotus leaf structure was measured using an arch-based reflection loss test system (Figure 3) at a frequency range of 18–26.5 GHz in an anechoic chamber. The compressive strength of the lotus leaf structure was measured using a universal test machine (E44, MTS, Huntsville, AL, USA) with a crosshead speed of 5.00 mm/min.

## 3. Results

### 3.1. Microstructure Characterization of Lotus Leaf Structure

**Figure 4:**
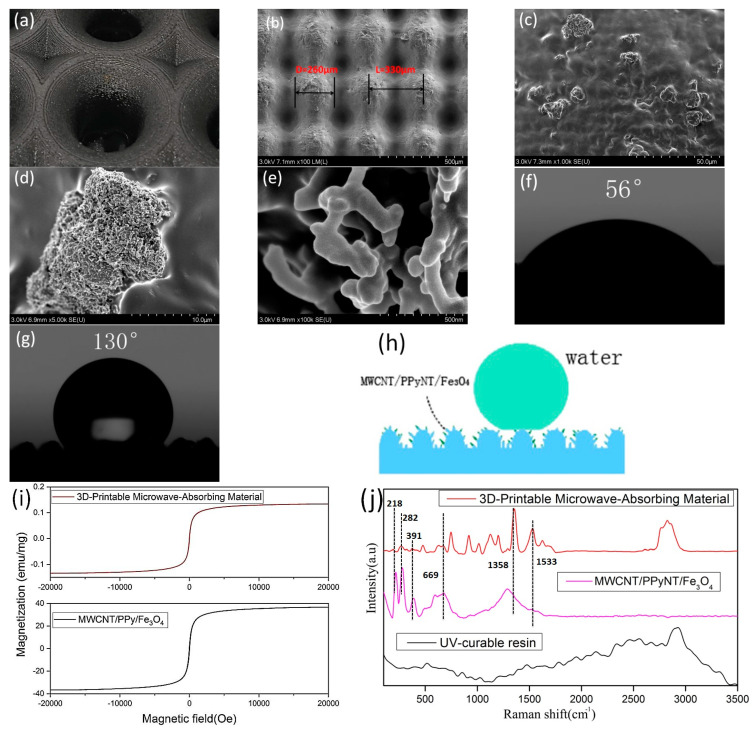
(**a**) Partial enlargement and (**b**–**e**) SEM images of the lotus leaf structure. (**f**,**g**) The static contact angle of water droplets on the surface of the absorbing material. (**h**) Schematic diagram of surface hydrophobicity. (**i**) Room temperature vibration sample magnetometry images of the 3D-printed microwave-absorbing material and MWCNT/PPy/Fe_3_O_4_ absorbers. (**j**) Raman spectra of the 3D-printed microwave-absorbing material, MWCNT/PPy/Fe_3_O_4_ absorbers, and UV-curable resins.

The performance of microwave absorbers in UV-curable resins and the design of the lotus leaf structures influence the absorption, hydrophobicity, and mechanical properties of 3D-printed microwave absorbers. First, the surface structure and electromagnetic parameters of the material were tested. As shown in Figure 4b, the diameter of the mastoid structure is D = 260 μm, and the distance between the mastoid structures is 220 μm. Based on the ratio between the actual size of the mastoid structure and the design size, the height of the mastoid is estimated to be approximately 350 μm. As shown in Figure 4c,d, there are protrusions with diameters ranging from 8 to 18 μm on the mastoid structure. Figure 4e shows that these protrusions form a network structure composed of MWCNT/PPy/Fe_3_O_4_ absorbers with an average diameter of 140 nm, which are exposed on the surface of the UV-cured resin. In addition, the micro/nanopapillae structure on the surface of the biomimetic lotus leaves can affect the wetting state of the material surface. The static contact angle of a water droplet on the surface of the radar wave-absorbing material without the printed papillae structure was found to be 56° (Figure 4f), which was measured using a contact angle measuring instrument. Furthermore, the static contact angle of water droplets on the surface of the absorbing material with the printed papillary structures was found to be 130°, as shown in Figure 4g. These tiny papillae structures form a large amount of air layer between them, which effectively reduces the contact area between the water droplets and the absorbing materials. This structure allows the water droplets to form spherical shapes on the surface of the lotus leaf structures, thereby making it difficult to wet the surface of the material (Figure 4h). Figure 4i shows the M–H hysteresis loops at room temperature for the 3D-printed microwave-absorbing material and MWCNT/PPy/Fe_3_O_4_ absorbers. The 3D-printed microwave-absorbing material demonstrates lower magnetic saturation strength, which can be attributed to its reduced Fe_3_O_4_ content. As shown in Figure 4j, the Raman spectrum of the 3D-printed microwave-absorbing material exhibits absorption peaks that are similar to those of MWCNT/PPy/Fe_3_O_4_ absorbers (i.e., 1358 cm^−1^ and 1533 cm^−1^, which correspond to the D and G peaks of the MWCNTs; the peak at 669 cm^−1^ corresponds to the E_g_ mode of Fe_3_O_4_; and the peak at 218 cm^−1^ is due to the A1g mode of α-Fe_2_O_3_ [38]). These findings indicate that the absorbent has stability during the curing process.

### 3.2. Electromagnetic Characterization of 3D-Printed Microwave-Absorbing Material

**Figure 5:**
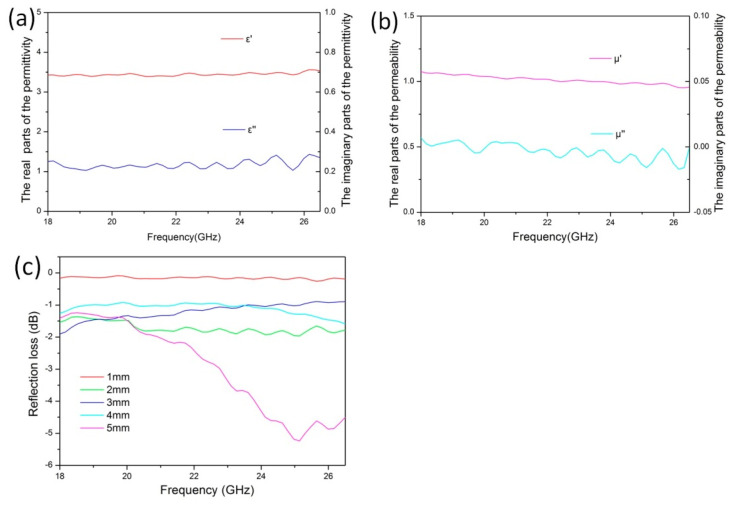
(**a**,**b**) Electromagnetic parameters and (**c**) electromagnetic loss property of the 3D-printed microwave-absorbing material in the K-band.

The electromagnetic properties of the 3D-printed microwave-absorbing material were measured. The corresponding results are shown in Figure 5. As can be seen, the average real part of the dielectric constant of the material is 3.5, and the average imaginary part is 0.25 (Figure 5a). In addition, the average real part of the magnetic loss is 1.1, and the imaginary part is approximately 0 (Figure 5b). The thickness of material is less than 4 mm, the electromagnetic loss does not exceed −2 dB, and when the thickness is 5 mm, the maximum electromagnetic loss is −5.2 dB at 25.1 GHz, as shown in Figure 5c.

### 3.3. Microwave Absorption Performance of Lotus Leaf Structure

**Figure 6:**
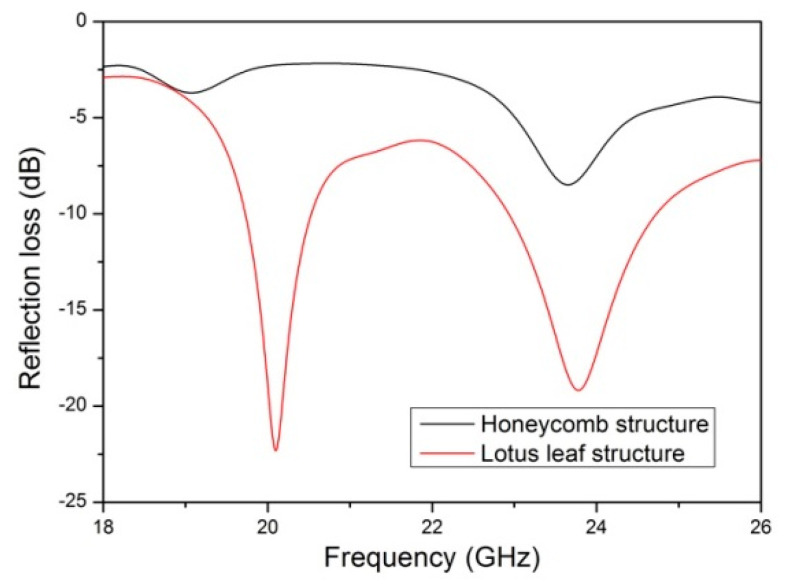
Simulated reflection loss for the lotus leaf and honeycomb structures.

The radar wave absorption performance of the lotus leaf structures in the K-band was optimized using the genetic algorithm in the CST Microwave Studio (2019) software. Periodic boundary conditions are applied in the x and y directions, whereas the z direction has an open boundary. A Floquet port is used as the excitation source to generate transverse electric and transverse magnetic plane waves. Additionally, a layer of total reflector is positioned below the lotus leaf structure. As shown in Figure 6, the lotus leaf structure has higher electromagnetic absorption performance than the honeycomb structure. The honeycomb structure prepared using the 3D-printed microwave-absorbing material has poor electromagnetic loss performance in the K-band, with a maximum absorption of −8.5 dB at 23.6 GHz. The biomimetic lotus leaf structure exhibits two strong absorption peaks in the K-band, with maximum absorption of −22.3 and −19.1 dB at 20.1 GHz and 23.8 GHz, respectively. In addition, the lotus leaf structure has a wide absorption wave band, with electromagnetic losses below −10 dB in the 19.7–20.5 GHz and 22.9–24.7 GHz wave bands. These simulation results indicate that the lotus leaf structure is suitable as a radar wave absorber in the K-band.

**Figure 7:**
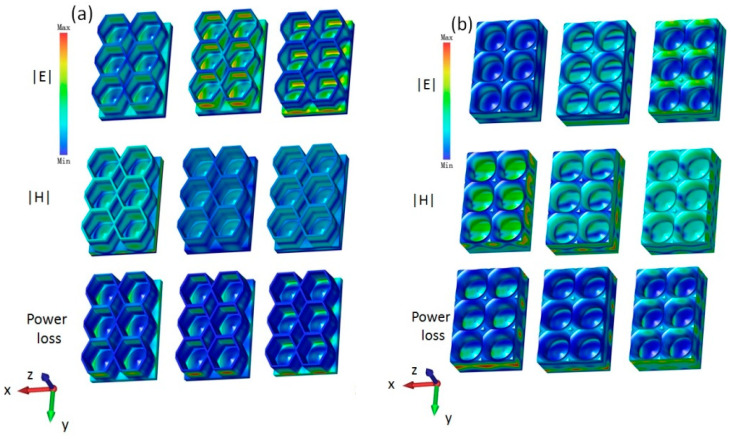
Electric field, magnetic field, and power loss density of the (**a**) honeycomb structure and (**b**) lotus leaf structure at 20 GHz, 22 GHz, and 24 GHz.

To determine the microwave absorption mechanisms of the lotus leaf and honeycomb structures, the distributions of the electric field intensity, magnetic field intensity, and power loss density were simulated at 20 GHz, 22 GHz, and 24 GHz. As shown in Figure 7a, the honeycomb structure is primarily characterized by electrical losses. Here, as the frequency increases, the intensity of the electrical losses increases, and the losses mainly occur on the bottom plate. Note that the magnetic loss of the honeycomb structure is relatively low, and its maximum energy and electrical losses are both at 24 GHz. Figure 7b shows that the magnetic loss of the lotus leaf structure is considerably higher than the electrical loss, with the maximum electrical loss at 26 GHz, which is primarily distributed along the Y-axis direction on the side walls. In addition, the maximum magnetic loss at 20 GHz is primarily concentrated on the bottom plate and the side walls along the X-axis direction. Thus, its energy loss shows two maximum values at 20 GHz and 26 GHz. Under the combined effect of the electromagnetic loss, the biomimetic lotus leaf structures have a wide microwave absorption band.

**Figure 8:**
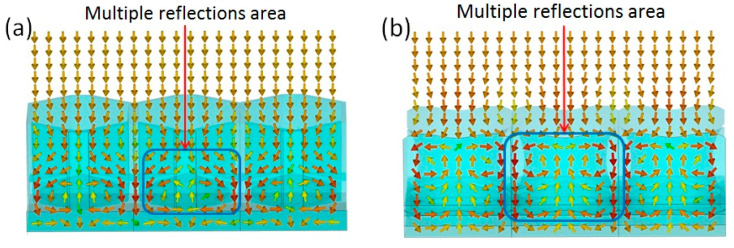
Power flow of honeycomb structure (**a**) and lotus leaf structure (**b**) at 20 GHz. The arrows in the figures are Poynting’s vectors.

The biomimetic lotus leaf structure has better loss characteristics in the K-band than the honeycomb structure. The honeycomb structure only has an electrical loss effect, and the lotus leaf structure has good electrical and magnetic loss performance. The electrical loss of the lotus leaf structure primarily plays a role in the high-frequency part, and the magnetic loss plays a role in the low-frequency part. Thus, it has two strong absorption peaks and a wide absorption band. To study the excellent electromagnetic loss characteristics of the lotus leaf structures, the CST Microwave Studio (2019) software was used to obtain the power flow at 20 GHz for both the honeycomb and lotus leaf structures. Figure 8a shows that the bottom of the honeycomb structure has a good antireflection structure, and the electromagnetic waves gradually flow toward the area between the honeycomb wall and the bottom cone, thereby forming a region with maximum energy loss. As shown in Figure 8b, due to the curvature of the side walls of the lotus leaf structure, the electromagnetic waves enter the interior of the structure, which are then guided to the area between the cone at the bottom of the cavity and the side walls. After multiple reflections, a great amount of the energy of the electromagnetic waves is lost. The lotus leaf structure has a more effective and larger antireflection area; thus, its electromagnetic loss performance is considerably better than that of the honeycomb structure.

**Figure 9:**
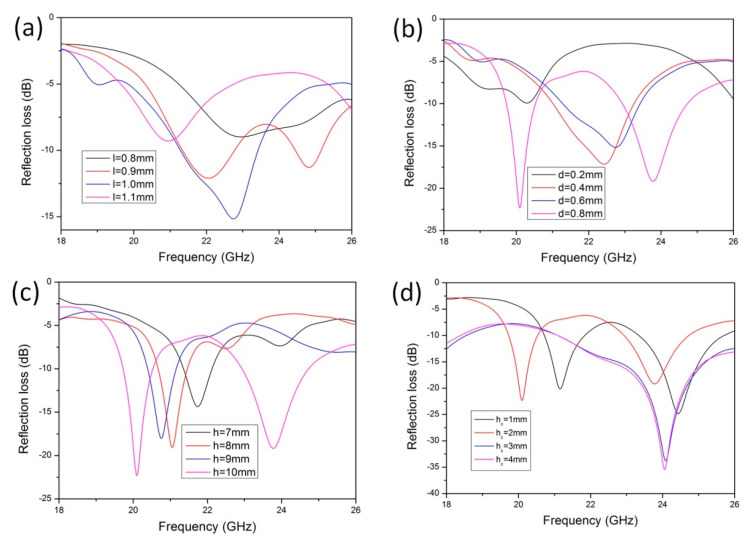
Geometry effects on the microwave absorption of the lotus leaf structures: (**a**) l, (**b**) d, (**c**) h, and (**d**) h_c_.

The lotus leaf structure plays a crucial role in the electromagnetic loss performance; thus, the influence of the geometric parameters of the lotus leaf structure on electromagnetic losses must be investigated. As shown in Figure 9a, with the increasing side length l of the lotus leaf structural unit, the electromagnetic loss performance initially increases and then decreases. When l = 1, there is a maximum value of −15.1 dB because the l value determines the plane size of the antireflection cavity. The l value determines the horizontal size of the antireflection cavity; thus, if the horizontal size of the unit is overly small or large, the number of reflections of radar waves in the cavity will decrease, which makes it easy to reflect from the cavity to the external space. Figure 9b shows that the electromagnetic absorption effect is best when the diameter of the narrowest part of the cavity’s antireflection pore structure (d) is 8 mm. At this time, the electromagnetic waves can easily enter the interior of the material. If the opening of the lotus leaf structure cavity is too small, the electromagnetic waves easily reflect back to the external space from the side wall. The higher the side wall of the lotus leaf structure (h), the larger the internal space of the antireflection chamber, which yields better electromagnetic loss performance (Figure 9c). When the height of the cone at the bottom of the lotus leaf structure is greater than 3 mm (h_c_), a better antireflection structure is obtained, and the electromagnetic loss value is higher, as shown in Figure 9d.

**Figure 10:**
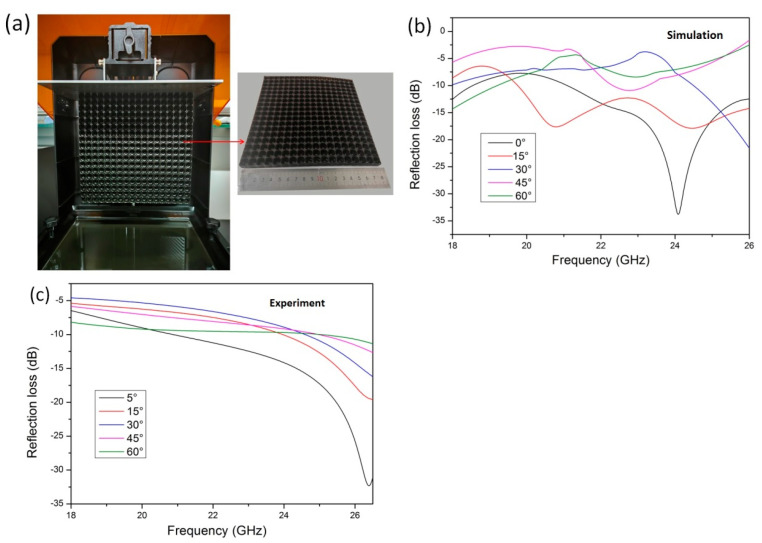
(**a**) Three-dimensionally printed lotus leaf structure radar wave absorber sample, (**b**) simulated reflection loss at different incident angles, and (**c**) measured reflection loss at different incident angles.

To verify the K-band absorption performance of the lotus leaf structure, the optimized geometric parameters (l = 1.0 mm, d = 0.8 mm, h = 10 mm, and h_c_ = 3 mm) were used to print electromagnetic loss test samples. Photographs of the samples are shown in Figure 10a. The radar wave absorption characteristics of the lotus leaf structure test samples were simulated and tested experimentally at different incident angles. The overall trend in the simulation results is similar to the experimental test results, and the difference in the results is primarily caused by geometry errors in the printed samples. As shown in Figure 10b,c, with the increasing incident angle, the electromagnetic loss in the K-band decreases gradually. The experimental test results demonstrate that the maximum electromagnetic loss is −32.3 dB when the incident angle is 5°, and the electromagnetic loss is below −10 dB in the 20.8–26.5 GHz band. The electromagnetic loss performance is better in the high-frequency region. Here, the maximum electromagnetic loss is −11.4 dB when the incident angle is 60°, and the absorption curve is relatively flat throughout the entire K-band. These results indicate that the electromagnetic absorption characteristics of the lotus leaf structure have a wide absorption band and incident angle in the K-band.

### 3.4. Mechanical Properties of Lotus Leaf Structure

**Figure 11:**
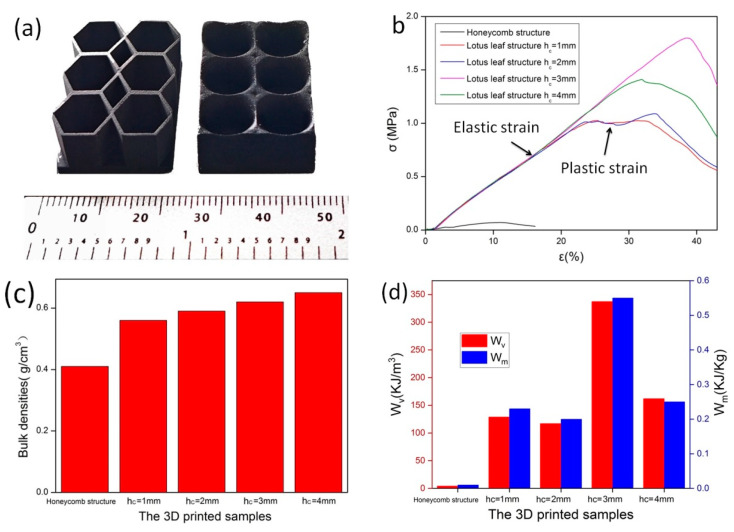
(**a**), Samples for the compressive strength test (**b**) stress–strain curves, (**c**) sample densities, and (**d**) mechanical energy absorption of the structures at different h_c_ values.

To evaluate the mechanical properties of the lotus leaf structure, 3 units × 2 units of samples were prepared, and their compressive strength was investigated (Figure 11a). Here, the mechanical properties of the lotus leaf and honeycomb structures with an average sidewall thickness of approximately 1 mm were compared, and the influence of the height h_c_ of the conical antireflection structure at the bottom of the lotus leaf cavity on its mechanical properties was evaluated. Figure 11b shows that the peak stress of the honeycomb structure is only 0.07 MPa, and the elastic strain is 12%. In contrast, the peak load of the lotus leaf structure is greater than 1 MPa. The sample with h_c_ = 3 mm has the highest peak stress of 1.80 MPa, and the elastic strain reaches 39%. When h_c_ = 1–2 mm, the plastic strain stage of the material is wider, exhibiting the characteristics of plastic materials. In addition, when the cone is low, the main support for the unit structure under compression is the sidewall, which can undergo plastic deformation. When h_c_ = 3–4 mm, an increase in the height of the cone provides support to the sidewall, which yields an increase in the peak stress of the material. However, the plastic deformation ability of the sidewall is reduced, and the plastic strain area shrinks, thereby exhibiting the characteristics of brittle materials.

Mechanical energy absorption is an important indicator when evaluating the mechanical properties of lightweight materials. The mechanical energy absorption per unit volume (W_V_) and the mechanical energy absorption per unit mass (W_m_) of a material during the elastic compression strain stage can be calculated using Equations (3) and (4) [39,40].

(3) $$W_{v}=\int_{0}^{\varepsilon_{E}}\sigma d\varepsilon$$

(4) $$W_{m}=W_{v}/\rho$$

Here, ρ is the bulk density of the structure, and $\varepsilon_{E}$ is the recoverable elastic strain [31]. Figure 11c shows the volumetric density of different structures, which is obtained by dividing the total mass of the material by the total volume. The honeycomb structure has a bulk density of 0.4 g/cm^3^, and that of the lotus leaf structure increases gradually from 0.56 g/cm^3^ to 0.65 g/cm^3^ with the height of the bottom cone. The W_V_ and W_m_ values of the lotus leaf structure are much greater than those of the honeycomb structure, as shown in Figure 11d. Here, the W_V_ and W_m_ values of the honeycomb structure are only 4.14 KJ/m^3^ and 0.01 KJ/Kg, respectively. For the lotus leaf structure, the maximum W_V_ and W_m_ values are 337.22 KJ/m^3^ and 0.55 KJ/Kg, respectively, when the cone height of the lotus leaf structure is 3 mm (h_c_ = 3 mm). These results demonstrate that the lotus leaf structure has good K-band radar wave absorption performance and good mechanical strength. The bottom cone of the lotus leaf structure can provide support for the side walls, and the height of the cone has a significant impact on the mechanical energy absorption performance of the material. Thus, the lotus leaf structure has better radar wave absorption ability and mechanical properties than commonly used honeycomb structures, and the lotus leaf structure is suitable as a lightweight and load-bearing K-band radar wave stealth component.

## 4. Conclusions

We added prepared multiwalled carbon nanotube/polypyrrole nanotube/Fe_3_O_4_ composites absorbent to UV-cured resin, and a novel lotus leaf-structured K-band radar wave absorber was prepared using a DLP 3D printer. We found that the microscale papillae structure printed on the surface of lotus leaf structure significantly improves the hydrophobicity of the material. In addition, the influence of the geometric parameter changes in the lotus leaf structure on the electromagnetic absorption characteristics and mechanical properties of the material was studied. After optimizing the geometric parameters of the lotus leaf structure, it exhibits absorption characteristics of broadband and wide-angle absorption in the K-band. The lotus leaf biomimetic structure designed in this study can be utilized as lightweight, hydrophobic, load-bearing, and radar wave-absorbing structure components in military equipment.

## Data Availability

The data presented in this paper are available on request from the corresponding author.
